# Association Between Statins Use and Major Bleeding in Patients Using Direct Oral Anticoagulants for Atrial Fibrillation

**DOI:** 10.1002/pds.70245

**Published:** 2025-10-25

**Authors:** A. de Burgos‐González, L. Cea‐Soriano, C. Huerta‐Álvarez

**Affiliations:** ^1^ Division of Pharmacoepidemiology and Pharmacovigilance Spanish Agency for Medicines and Medical Devices (AEMPS) Madrid Spain; ^2^ Department of Public Health and Maternal Child Health, Faculty of Medicine Complutense University of Madrid Madrid Spain

**Keywords:** DOAC, major bleeding, nested case control study, statins

## Abstract

**Background:**

Direct oral anticoagulants (DOAC) were developed as an alternative to vitamin K antagonists in the treatment of NVAF. Statins are frequently prescribed drugs for the prevention of atherosclerotic cardiovascular disease. A potential interaction between DOACs and statins has been described, suggesting that their concomitant use may increase the risk of major bleeding (MB).

**Purpose:**

To estimate the risk of MB associated with concomitant use of DOACs and statins to assess potential safety issues.

**Methods:**

A case–control study nested in a cohort of new users of DOACs was performed in BIFAP. Cases were defined as MB events and matched to four controls by risk‐set sampling. Treatment episodes were built to define the participants' exposure according to their use at index date as current, recent, past, or no use. Exposure was also assessed in relation to its continuity and duration. Variables were created based on the concomitant use of DOACs and statins to study the interaction, using conditional logistic regression to estimate the risk of MB.

**Results:**

aOR (95% CI) for current use of DOAC or statin use were 1.35 (0.96–1.9) and 0.72 (0.59–0.88). aOR for their concomitant use was 1.31 (0.83–2.07) compared to nonuse of either of them. DOAC continuous use (1.27; 0.8–2.03) and long‐term (1.11; 0.67–1.81) treatment was associated with smaller risks than noncontinuous (1.40; 0.85–2.32) and short‐term treatment (1.43; 0.94–2.45). Analyses by individual DOACs and statins showed similar results except for edoxaban, which had low numbers.

**Conclusions:**

There was no evidence to suggest an interaction between DOACs and statins and the risk of MB. Regular use of DOACs is important to minimize their impact on MB onset.

**Summary**

- This study investigates the real‐world safety of concomitant use of DOACs and statins using a large, representative EHR database from Spain (BIFAP).
- We applied a nested case–control design within a cohort of patients initiating DOAC treatment for NVAF, minimizing confounding by indication.
- Results showed a nonsignificant elevated risk of MB associated with the use of anticoagulants regardless of statin use.
- Findings suggest that concomitant use of DOAC and statins does not increase the risk of major bleeding, regardless of continuous use or treatment duration.
- Individual analyses of DOACs (dabigatran, apixaban, rivaroxaban) and statins (atorvastatin, simvastatin/lovastatin) showed consistent, similar results.
- Results highlight the importance of treatment adherence and provide reassurance for clinicians prescribing these commonly coadministered drugs.

## 1 Introduction

Direct oral anticoagulants (DOACs) are a group of anticoagulants used to reduce the risk of stroke and systemic embolism in patients with non‐valvular atrial fibrillation (NVAF) [1, 2]. They were developed as an alternative to vitamin K antagonists, and studies have shown that they are as safe and effective in the prevention of stroke in patients with NVAF. Moreover, DOACs have a wider therapeutic range than warfarin, allowing them to avoid routine blood tests [3, 4], which has enhanced their use as common anticoagulant drugs [5, 6, 7].

Previous studies have investigated drug–drug interactions of DOACs. In one study [8], authors found that nearly half of the patients with major bleeding (MB) used a drug that can potentially cause a pharmacokinetic interaction with DOACs, with simvastatin, atorvastatin, and digoxin being the most commonly coadministered interacting drugs. Statins are among the most prescribed drugs worldwide, as they are indicated in the prevention of atherosclerotic cardiovascular disease [9, 10]. Thus, the potential for interaction of these drugs must be considered, especially in elderly patients with comorbidities and polypharmacy [11].

Pharmacokinetic mechanisms of such potential interaction have been described [12, 13]. DOACs are substrates of the transport protein P‐glycoprotein (P‐gp), which is known to affect the bioavailability of many drugs. Atorvastatin, lovastatin, and simvastatin may inhibit or compete with P‐gp, increasing absorption and exposure to DOACs, resulting in an increased risk of bleeding. Apixaban, edoxaban, and rivaroxaban are metabolized in the liver by the cytochrome P450 3A4 system (CYP3A4); therefore, they are also susceptible to alterations in this hepatic cytochrome, influencing their pharmacological effects [14]. Atorvastatin, lovastatin, and simvastatin are inducers of CYP3A4, which increases the metabolism of some DOACs, leading to a prothrombotic effect. Dabigatran is not metabolized by the cytochrome P450 3A4 system and is directly excreted by the kidneys, but its plasma concentration is modulated by P‐gp [14].

Observational studies on this topic [15] showed a higher risk of MB in patients using concomitant dabigatran and simvastatin or lovastatin compared to dabigatran alone. However, opposite results were also found [16].

Real‐world data studies focusing specifically on this interaction are scarce, and results are controversial with no specific information on all individual DOACs [14]. In order to fill major gaps, we performed a case–control study nested in a cohort of DOACs users for NVAF in an Electronic Health Records (EHR) database in Spain, with the aim of evaluating whether coadministration of statins and DOACs increases the occurrence of MB.

## 2 Methods

We performed a case–control study nested within a cohort of patients with a first prescription of a DOAC for NVAF between 2008 and 2018.

### 2.1 Data Source

The study was conducted in BIFAP, a longitudinal population‐based database, funded by AEMPS and containing anonymized EHR from patients attended by primary care practitioners and pediatricians (PCP) belonging to the National Health System. Nine Autonomous regions collaborate by providing data. Information available in BIFAP includes patient demographics, lifestyle factors, clinical events, electronic prescriptions, and dispensations in pharmacies. BIFAP uses ICD‐9 and ICPC‐2 terminologies. At the time of this study, BIFAP included EHRs of approximately 12 million patients, covering almost 25% of the Spanish population, with an average follow‐up of 8.5 years. The BIFAP population was representative of the Spanish population in terms of age and sex. BIFAP has been extensively described elsewhere [17].

### 2.2 Study Population and Setting

To conduct the case–control study nested in a cohort of new users of DOACs (defined as 1 year without any DOAC use before the study entry date), we first selected a cohort of patients aged 18 years and older, with at least 1 year of registration with the PCP between 2008 and 2018, and a DOAC prescription for NVAF (Figure S1). The first recorded DOAC prescription date was considered the study entry date. From this date, new users of DOACs were followed up until the occurrence of the following endpoints: MB, death, end of the study period, or exit from the database, whichever came first. The date of the MB was considered the index date (ID). Patients were excluded if their ID was the same as their study entry date.

All patients with an MB event, defined as an adapted version of the definition of the International Society on Thrombosis and Hemostasis [18], during the study period were considered cases. Validation of MB has been fully explained elsewhere [19].

A set of controls was randomly chosen from the cohort of DOAC new users by incidence density sampling (risk‐set sampling), meaning that controls were identified from patients in the cohort who were “at risk” at the ID of a case. This is a sampling with replacement method, so that a patient could be selected as a control for different cases and cases could be controls before having the event of interest. This nested case–control design is a valid approach in pharmacoepidemiologic studies of this type, as incidence density sampling provides unbiased estimates of relative risk equivalent to those obtained from a full cohort analysis [20, 21, 22]. Case–control ratio was 1:4, matched by age (±2 years), sex, and autonomous region of origin.

### 2.3 Exposure Definition

To assess exposure, DOAC (as a group and by individual DOAC including dabigatran, rivaroxaban, apixaban, and edoxaban) and statins (including simvastatin, lovastatin, and atorvastatin) treatment episodes of the patients were built. A treatment episode was defined as the period from the first recorded prescription of a drug until the patient stopped using it, considering the end of treatment as a distance or gap between prescriptions of more than 30 days. Based on the treatment episodes, patients were classified according to the type of use on the ID using the following categories: current use, when the supply of the most recent prescription lasted until the ID or ended in the 30 days before; recent use, when it lasted until 30–90 days before the ID; past use, when it lasted until 90–365 days before the ID; and no use, when the most recent prescription ended more than 365 days before the ID. Furthermore, current users were classified as continuous users if they had only one treatment episode (the first episode) throughout the study period. In addition, among current users, the duration of treatment was calculated by adding individual durations of consecutive prescriptions, and it was classified as short‐term (≤ 365 days) or long‐term (> 365 days).

To study interaction, we created variables based on the concomitant use of DOACs and statins. Each patient can be classified as current concomitant users of DOACs and statins (A + B), current users of only DOACs (A + No B), or only statins (No A + B), no use of either drug (No A + No B), or other possible combinations. These interaction variables were created for all individual DOACs. Statins were divided into lovastatin/simvastatin as a group and atorvastatin, based on pharmacokinetic differences in their effect on P‐gp and CYP3A4 inhibition, which might lead to an increased absorption or metabolism of DOACs [12, 14, 23]. In addition, a CYP3A4 moderate inhibitor effect has only been described for atorvastatin.

### 2.4 Statistical Analysis

The study population was described by differentiating between cases and controls. Some variables were categorized for a better description, including “missing” values as one more category for BMI, creatinine, alcohol, and tobacco.

Case–control analyses were conducted using conditional logistic regression to calculate ORs with 95% confidence intervals (95% CI) as a measure of the relative risk of MB associated with concomitant use of DOACs and statins. To adjust the model, standardized differences (SMD) were used to compare the baseline covariates between cases and controls; an SMD smaller than 10% indicated a good balance for a given covariate [24]. It was calculated as the difference in the proportions between the two groups in units of pooled standard deviation. Vitamin K antagonists were specifically included in the model. Variables included in the analysis model were diabetes, previous bleeding, anemia, kidney disease, hepatic disease, heart failure, peripheral vascular disease, cancer, use of antiplatelet drugs, corticosteroids, diuretics, histamine inhibitors, and vitamin K antagonists, as well as BMI, creatinine, and dyslipidemia.

A sensitivity analysis was performed to test the definition of current use. An exposure time window and gap period of 7 days instead of 30 days was used to ensure that the use of DOACs and statins really overlapped.

## 3 Results

### 3.1 Baseline Characteristics of Cases and Controls

A total of 59 461 patients comprised the study population of new users of DOACs for NVAF. There were 923 MB cases matched to 3692 controls (Figure S1). Table 1 describes baseline characteristics of cases and controls. The mean age of the study population was 81 years, and 53% were males. The most prevalent comorbidities were hypertension (75%), dyslipidemia (46%), and mental disorders (56%). Antihypertensive drugs (86%) and proton pump inhibitors (59%) were mostly prescribed.

**TABLE 1:** Baseline characteristics of study population among cases and controls.

	Controls (*n* = 3692)		Cases (*n* = 923)		SMD
	*n*	%	*n*	%	
*Sex*					
Male	1972	53.4	493	53.4	0
*Age*					
Mean age (years, SD)	80.96	8.28	81.09	8.33	1.52
< 65	162	4.4	41	4.4	0.26
65–75	561	15.2	135	14.6	1.6
75–85	1605	43.5	400	43.3	0.27
> 85	1364	36.9	347	37.6	1.34
*GP visits during study period*					
Mean (SD)	21.48	15.55	25.86	3.17	26.59
< 5	233	6.3	20	2.2	20.68
≥ 5 and < 10	524	14.2	92	10.0	12.99
≥ 10 and < 15	652	17.7	132	14.3	9.18
≥ 15 and < 20	605	16.4	155	16.8	1.09
≥ 20 and < 30	849	23.0	232	25.1	5.01
≥ 30	829	22.5	292	31.6	20.78
*BMI*					
< 18.5	23	0.6	8	0.9	2.84
18.5–24.9	541	14.7	146	15.8	3.24
25–29.9	1312	35.5	299	32.4	6.64
30–34.9	843	22.8	204	22.1	1.75
35–39.9	242	6.6	57	6.2	1.55
> 40	80	2.2	32	3.5	7.86
Unknown	651	17.6	177	19.2	3.99
*Alcohol*					
Alcohol consumption	815	22.1	231	25.0	6.96
No alcohol consumption	1724	46.7	428	46.4	0.65
Unknown	1153	31.2	264	28.6	5.74
*Creatinine*					
Low	82	2.2	21	2.3	0.37
Normal	1431	38.8	343	37.2	3.29
High	336	9.1	109	11.8	8.86
Unknown	1843	49.9	450	48.8	2.33
*Tobacco*					
Nonsmoker	1383	37.5	337	36.5	1.96
Former smoker	262	7.1	77	8.3	4.67
Smoker	344	9.3	96	10.4	3.64
Unknown	1703	46.1	413	44.7	2.78
*Comorbidities*					
Hypertension	2749	74.5	720	78.0	8.34
Dyslipidemia	1710	46.3	433	46.9	1.2
Diabetes	1002	27.1	304	32.9	12.67
Intracranial bleeding	54	1.5	27	2.9	10
Bleeding	389	10.5	167	18.1	21.7
TIA	278	7.5	71	7.7	0.61
Stroke	412	11.2	119	12.9	5.33
VTE	126	3.4	41	4.4	5.3
Aneurism	82	2.2	23	2.5	1.79
Anemia	931	25.2	321	34.8	20,98
Coagulation disorders	322	8.7	79	8.6	0.58
Thrombocytopenia	385	10.4	99	10.7	0.97
Renal disease	539	14.6	179	19.4	12.79
Hepatic disease	94	0.3	49	5.3	14.26
Digestive disease	998	27.0	286	31.0	8.72
Heart failure	711	19.3	235	25.5	14.93
Coronary artery disease	212	5.7	52	5.6	0.47
Malignancy	512	13.9	179	19.4	14.88
Asthma/COPD	693	18.8	198	21.5	6.69
Mental disorders	2054	55.6	526	57.0	2.73
Epilepsy	71	1.9	14	1.5	3.13
PVD	199	5.4	76	8.2	11.31
Fracture	752	20.4	223	24.2	9.12
*Drug use* ^a^					
Antiplatelet agents	424	11.5	165	17.9	18.14
Clopidogrel	72	2.0	40	4.3	13.7
ASA	339	9.2	129	14.0	15.03
Tramadol	70	1.9	25	2.7	5.42
Paracetamol	1159	31.4	331	35.9	9.47
NSAIDs	434	11.8	121	13.1	4.11
Corticosteroids	226	6.1	90	9.8	13.46
Antihypertensive drugs	3168	85.8	822	89.1	9.82
Diuretics	1628	44.1	474	51.4	14.57
RAS‐acting agents	2020	54.7	507	54.9	0.44
Digoxin	496	13.4	129	14.0	1.58
PPIs	2138	57.9	563	61.0	6.29
H2‐blockers	83	2.2	39	4.2	11.19
Oral hypoglycemic drugs	744	20.2	185	20.0	0.27
Insulins	212	5.7	75	8.1	9.39
Antiepileptic	257	7.0	88	9.5	9.36
Antidepressants	750	20.3	195	21.1	2.01
Anxiolytic	971	26.3	271	29.4	6.83
VKAs	235	6.4	96	10.4	14.6

Abbreviations: ASA, acetylsalicylic acid; BMI, body mass index; COPD, chronic obstructive pulmonary disease; GP, general practitioner; H2‐blockers, histamine H2‐receptor antagonists; NSAIDs, nonsteroidal anti‐inflammatory drugs; PPIs, proton pump inhibitors; RAS‐acting agents, agents acting on the renin–angiotensin system; SD, standard deviation; SMD, standardized mean difference; TIA, transient ischemic attack; VKAs, vitamin K antagonists; VTE, venous thromboembolism disease.

^a^ Drug use in the 90 days prior to index date.

### 3.2 DOACs, Statins, and Risk of MB

**FIGURE 1:**
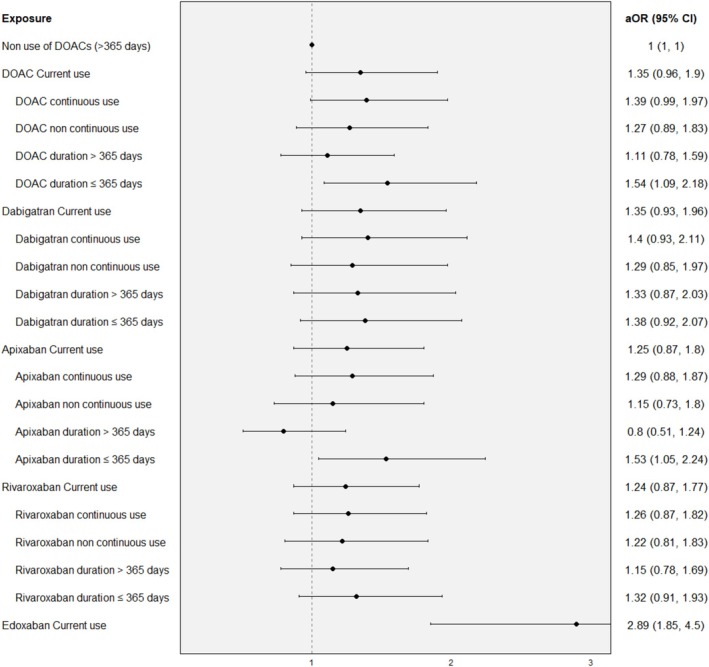
Association between use of DOACs and the risk of major bleeding according to type of current use and treatment duration.

**FIGURE 2:**
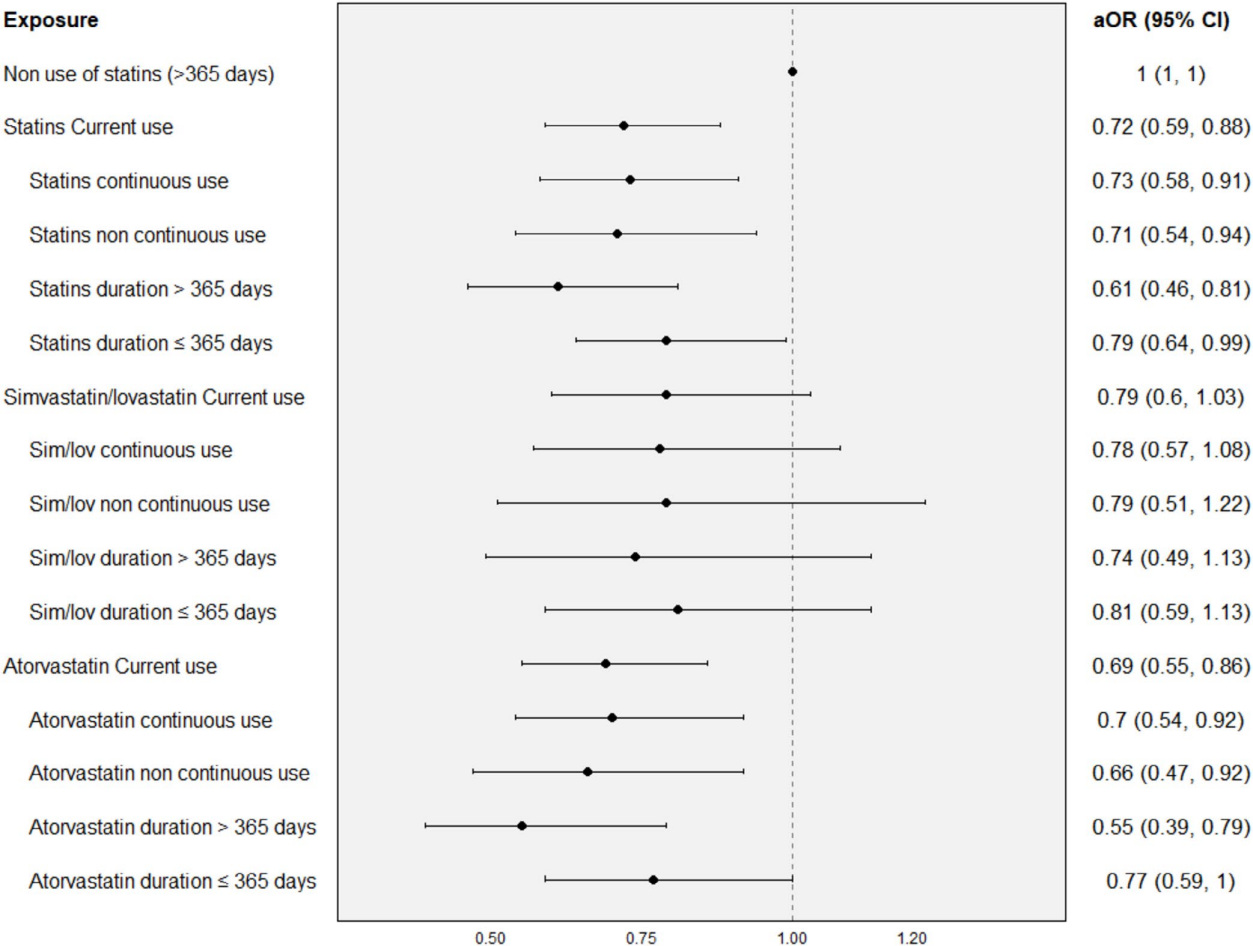
Association between use of statins and the risk of major bleeding according to type of current use and treatment duration.

Figures 1 and 2 show the individual effects of DOACs and statins separately on MB risk onset. Current use of DOACs showed an aOR of 1.35 (95% CI: 0.96–1.9), with slightly higher risk for continuous use than noncontinuous use (aOR of 1.39 (95% CI: 0.99–1.97) and 1.27 (95% CI: 0.89–1.83), respectively) and considerably higher risk for short‐term treatment than long‐term (aOR of 1.54 (95% CI: 1.09–2.18) and 1.11 (95% CI: 0.78–1.59), respectively). Risk was even higher for short‐term treatment than for long‐term among continuous users of DOACs (Figure S2). Individual DOACs presented similar risk estimates, except for edoxaban, which showed an aOR of 2.89 (95% CI: 1.85–4.5) although this result should be interpreted with caution due to low numbers. We also observed similar risk patterns for individual DOACs when comparing continuous and noncontinuous use and treatment duration. Notably, these specific analyses were not performed for edoxaban because of their very low numbers.

Statin current use showed a protective effect on MB risk compared with nonuse (aOR of 0.72, 95% CI: 0.59–0.88). Noncontinuous use had a similar aOR to continuous use (aOR of 0.71 (95% CI: 0.54–0.94) vs. 0.73 (95% CI: 0.58–0.91)), and long‐term treatment had a smaller aOR than short‐term (aOR of 0.61 (95% CI: 0.46–0.81) vs. 0.79 (95% CI: 0.64–0.99)). Analysis of atorvastatin showed a smaller MB risk (aOR of 0.69, 95% CI: 0.55–0.86) than simvastatin and lovastatin (0.79, 95% CI: 0.6–1.03). Continuous use and treatment episode duration for individual statins showed similar trends as the overall statin analysis (Figure S2).

### 3.3 Interaction Between DOACs and Statins

**FIGURE 3:**
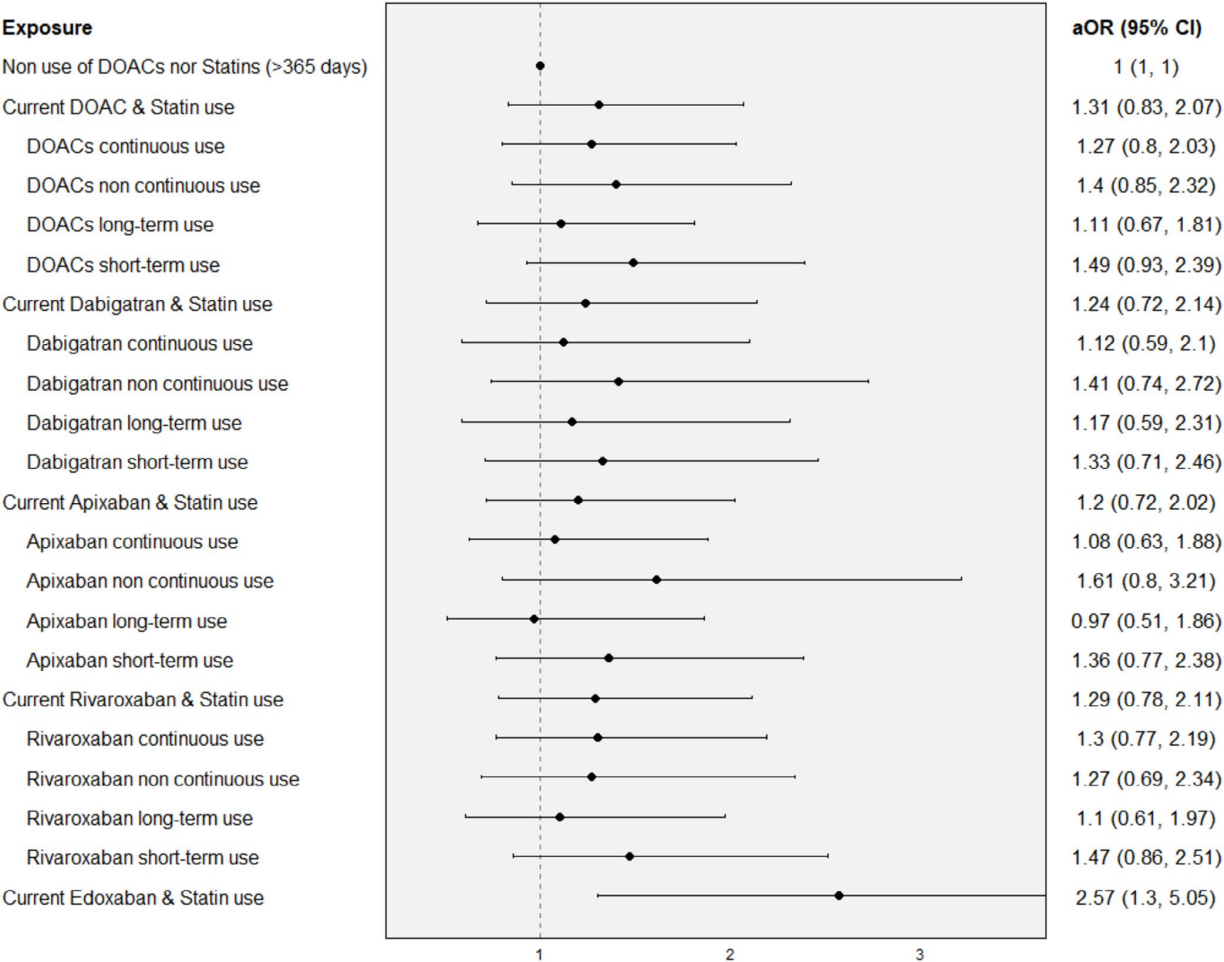
Association between the concomitant use of DOACs and statins and the risk of major bleeding according to type of current use and treatment duration.

Interaction analysis showed that, compared with nonuse of either DOACs or statins, the risk of current use of both drugs yielded an estimate of 1.31 (95% CI: 0.83–2.07) (Figure 3). When evaluating the type of current DOAC use in the interaction, the risk was smaller for patients with continuous use of DOACs compared with noncontinuous use (aOR of 1.27 (95% CI: 0.8–2.03) and 1.40 (95% CI: 0.85–2.32), respectively). When studying the effect of DOAC treatment duration, the risk of MB was 1.11 (95% CI: 0.67–1.81) for long‐term use and 1.43 (95% CI: 0.94–2.45) for short‐term. When stratifying treatment duration according to continuous use, risk was even higher for short‐term than long‐term among continuous users (Figure S3).

### 3.4 Interaction Between Individual DOACs and Statins

Analysis by individual DOAC (Figure 3) also showed nonsignificant estimates associated with the concomitant use of each type of DOAC and statins (aOR of 1.24 (95% CI: 0.72–2.13) for dabigatran, 1.20 (95% CI: 0.72–2.02) for apixaban, and 1.29 (95% CI: 0.78–2.11) for rivaroxaban), except for edoxaban (aOR of 2.47, 95% CI: 1.26–4.86), as seen in the individual analysis. Continuous use showed a smaller risk than noncontinuous use for dabigatran and apixaban, and slightly higher for rivaroxaban. Treatment episode duration showed consistently smaller risk for long‐term use for each individual DOAC, also among continuous users (Figure S3).

### 3.5 Interaction Between DOACs and Individual Statins

The aORs associated with concomitant use of DOAC and simvastatin/lovastatin were 1.48 (95% CI: 0.9–2.43) and 1.22 (95% CI: 0.76–1.96) for atorvastatin (Figures S4 and S5). Analysis by individual DOACs showed some differences between concomitant use of each statin, but there were no significant risks in any category (Figures S4 and S5).

Concomitant use of atorvastatin with DOACs showed smaller risks for continuous use than for noncontinuous. However, the opposite trend was found for concomitant use of simvastatin or lovastatin with DOACs, showing a higher risk for continuous users (except for apixaban). Short‐term DOAC treatment showed higher risks than long‐term for every combination of DOACs and statins, except for concomitant rivaroxaban and simvastatin or lovastatin use (Figure S6).

### 3.6 Sensitivity Analysis

Using a gap of 7 days for the definition of current use showed similar results, with an aOR of MB for the concomitant use of DOACs and statins of 1.23 (95% CI: 0.78–1.94). Individual DOACs' risks were as follows: 1.21 (95% CI: 0.7–2.11) for dabigatran, 1.10 (95% CI: 0.65–1.86) for apixaban, 1.19 (95% CI: 0.72–1.97) for rivaroxaban, and 2.31 (95% CI: 1.15–4.63) for edoxaban (Figure S7).

## 4 Discussion

This study aimed to evaluate potential pharmacokinetic interactions between the concomitant use of DOACs and statins. We did not find any potential interaction on the increase in the risk of MB when both drugs were used concomitantly. This was also observed regardless of the type of current concomitant use and treatment duration. There was a consistent nonsignificant elevated risk of MB associated with the use of anticoagulants (aOR of 1.35; 0.96–1.9) regardless of statin use, which is expected when prescribing these drugs [25], considering the target population in terms of age and comorbidities. However, we found that the concomitant use of statins (aOR of 1.31; 0.83–2.07) does not modify this risk, indicating that there may be no interaction between these two drugs.

Only a few studies have evaluated this potential interaction in real‐world settings. A study found that the use of dabigatran concomitantly with simvastatin and lovastatin was associated with an increased risk of MB (OR 1.46, 95% CI: 1.17–1.82) [15]. This study generated a signal from the European Medicines Agency to study this interaction [26]. Subsequent studies found very broad results, ranging from an increased risk [27] of MB associated with concomitant DOACs and statin use to no clinically significant interaction [8, 28] and even a reduced incidence of MB [16, 29, 30] when using these drugs together. Also, results from two small trials [31, 32] found low interaction potential between dabigatran and atorvastatin or simvastatin. All these studies had considerable differences in their design, outcome definition, and methodology of analysis, making comparisons difficult, although no interaction between DOACs and statins appeared to be the most common conclusion. Prior studies have used different definitions when choosing the reference group; the current study has applied neither use of DOACs nor statins, providing an additional point of view to the assessment of exposure to these drugs.

Individual analysis of the three most prevalent DOACs (dabigatran, apixaban, and rivaroxaban) showed nonsignificant risk estimates, both as individual exposure and with concomitant use of statins, emphasizing that the concomitant use of both drugs (DOACs and statins) barely modified the risk of MB in these patients. This result was supported by interaction analysis results with individual statins, which showed negligible differences in the risk estimates for simvastatin/lovastatin and atorvastatin. Contrary to these results, edoxaban yielded a higher MB risk. This may be explained by confounding by indication because, at the time of the study, edoxaban was the newest marketed DOAC, which may suggest that it was specially prescribed to patients who had previously failed other available treatments.

We explored the continuous use and duration of the treatment episodes and found a general trend towards higher MB risks for noncontinuous than continuous users and a stronger one for short‐term treatments compared with long‐term, as seen in other studies [33]. These findings suggest the importance of anticoagulant treatment adherence to avoid unnecessary adverse events [34, 35], especially in older and vulnerable populations. These analyses provide context on how adherence patterns, particularly short‐term and noncontinuous use, may influence the estimates of concomitant DOAC–statin use, without aiming to draw new conclusions on adherence itself.

A key strength of this study was the inclusion of a study population based on the real population, which makes our results generalizable and a reflection of clinical practice in the primary care setting. The database also contains complete information on the prescription of the drugs of interest, which makes the estimation of their use and treatment patterns very reliable. The definition of a 30‐day gap for building treatment episodes has been used and found appropriate in previous studies on DOACs performed in BIFAP [36]; however, a sensitivity analysis using a 7‐day gap helped us confirm that our estimations were not affected by a broad assumption of the use of DOACs. Indication bias was controlled by including only patients with NVAF, which was found both as a direct link from the DOAC prescription for the patient and a search for NVAF fibrillation diagnosis in the patient history, excluding those with a previous history of valvular diseases.

One potential limitation of this study could be that, although the number of MB cases provided adequate power for the main analyses, estimates from some stratified analyses (e.g., by individual DOACs or statins) should be interpreted with caution due to limited sample size and wide confidence intervals. Also, BIFAP includes PCP prescription and/or dispensed anticoagulants, which do not necessarily mean the actual use of the drug, something common to every secondary database study. However, this is not expected to be very likely, as subjects repeatedly picked up the prescriptions. In BIFAP, information on inpatient and specialist prescribing is not available; however, only PCP‐prescribed anticoagulant dispensing had been shown not to materially impact the effect estimates compared with including all anticoagulant dispensing [37]. Prescriptions from private doctors are not included; however, it is expected not to impact the estimates, as those drugs are under the reimbursement system in Spain, so it is assumed that most prescriptions are issued in the public health system. We evaluated a cohort of new users of DOACs to minimize residual confounding, but this cannot be entirely ruled out. Another limitation includes not studying the dose of each DOAC, as it may influence the risk of MB from anticoagulants and explain some of the differences in the risk estimates found between individual DOACs [38]. Finally, clinically relevant nonmajor bleeding (CRNMB) may contribute to treatment discontinuation and explain differences between continuous and noncontinuous users, but models were adjusted for previous bleeding since CRNMB is not explicitly recorded in BIFAP; nonetheless, CRNMB deserves attention in future research given its clinical impact.

In conclusion, this study did not find a clinically relevant interaction between DOACs and statins in the risk of MB based on real‐world data. Results from continuous use and treatment duration of DOACs showed that the regular use of DOAC therapy is important to minimize the impact of these drugs on MB onset. Nevertheless, considering the controversy across this topic and the changes in DOAC drug utilization patterns over the last decade, further investigation is warranted.

### 4.1 Plain Language Summary

DOACs are commonly used to prevent strokes in people with NVAF. Statins, a drug indicated to protect against heart disease, are also frequently prescribed to these patients. Some studies described the possibility that statins may affect the safety of DOACs when used together and increase the risk of MB. To better understand this potential risk, we used data from EHRs from Spain to perform a nested case–control study, using conditional logistic regression models to estimate the risk of MB of people taking DOACs and/or statins with those that did not. We found that the use of DOACs had a slight risk of MB that did not increase when also using statins. This pattern was found regardless of the type of DOAC used, except for edoxaban, for which there was not enough data. We also found that MB risk associated with DOAC use was smaller when using them continuously and for longer terms. These findings suggest that there is no increased risk of MB when using statins alongside DOACs.

## Author Contributions

C. Huerta‐Álvarez originated and designed the study. A. de Burgos‐González and L. Cea‐Soriano collected data and contributed to the analysis. A. de Burgos, C. Huerta‐Álvarez, and L. Cea‐Soriano contributed to the interpretation of the results and to the drafting of the paper. All authors contributed to and approved the final version of the article and the authorship list.

## Ethics Statement

This study was approved by the Scientific BIFAP Committee (Reference #13_2017).

## Conflicts of Interest

The authors declare no conflicts of interest.

## Supporting information


**Figure S1:** Flowchart of the study.

**Figure S2:** Association between the use of DOACs or statins and the risk of major bleeding according to treatment duration, stratified by type of current use.

**Figure S3:** Association between the concomitant use of DOACs and statins and the risk of major bleeding according to treatment duration, stratified by type of current use.

**Figure S4:** Association between the concomitant use of DOACs and simvastatin/lovastatin or atorvastatin and the risk of major bleeding according to type of current use.

**Figure S5:** Association between the concomitant use of DOACs and simvastatin/lovastatin or atorvastatin and the risk of major bleeding according to treatment duration.

**Figure S6:** Association between the concomitant use of DOACs and simvastatin/lovastatin or atorvastatin and the risk of major bleeding according to treatment duration, stratified by type of current use.

**Figure S7:** Sensitivity analysis: Association between the concomitant use of DOACs and statins and the risk of major bleeding according to current use definition of 7 days.

## Data Availability

C. Huerta‐Álvarez remains custodian of the individual patient‐level health information. Due to data protection regulations, individual‐level data from this study and administrative registries cannot be shared by the authors, and requests to access the Spanish datasets should be directed to http://www.bifap.org/.
