# Supplementary figures and images for: Association Between Statins Use and Major Bleeding in Patients Using Direct Oral Anticoagulants for Atrial Fibrillation
Source: Pharmacoepidemiol Drug Saf. 2025 Oct 25;34(11):e70245. doi: 10.1002/pds.70245 (PMC12553355; doi:10.1002/pds.70245)

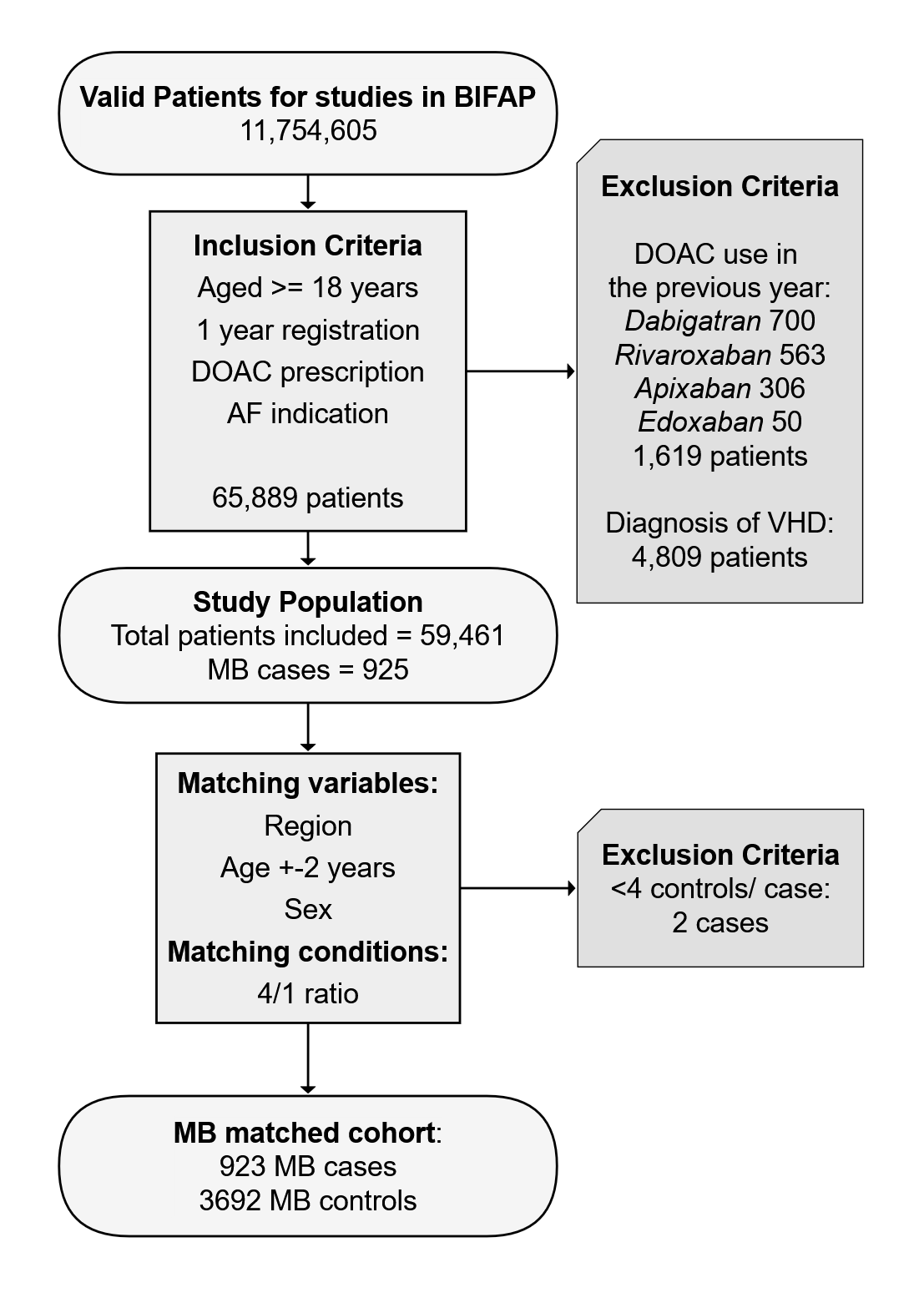

Supplement: Supplementary file 1 — Figure S1: Flowchart of the study. [file PDS-34-e70245-s004.png]

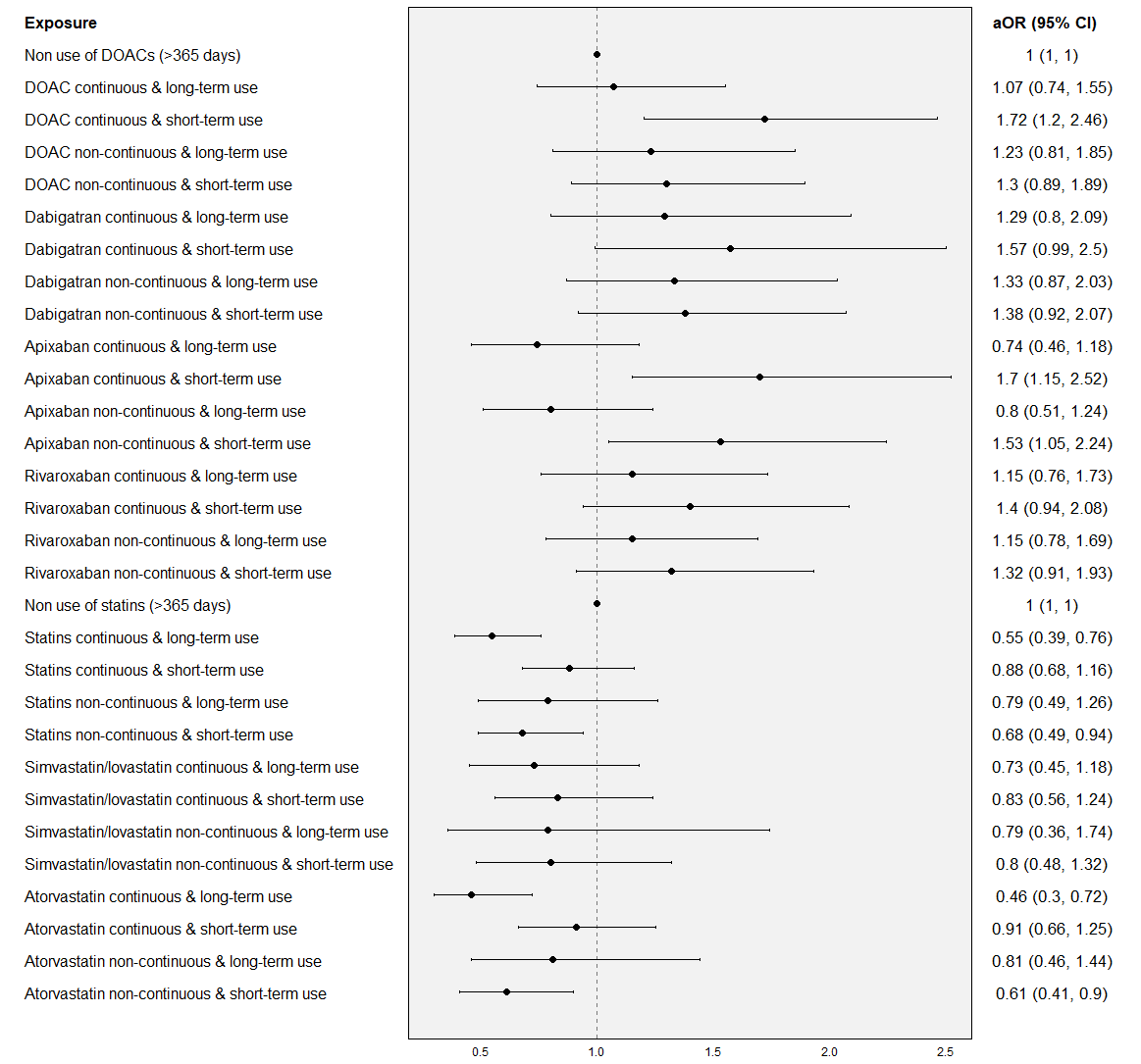

Supplement: Supplementary file 2 — Figure S2: Association between the use of DOACs or statins and the risk of major bleeding according to treatment duration, stratified by type of current use. [file PDS-34-e70245-s005.png]

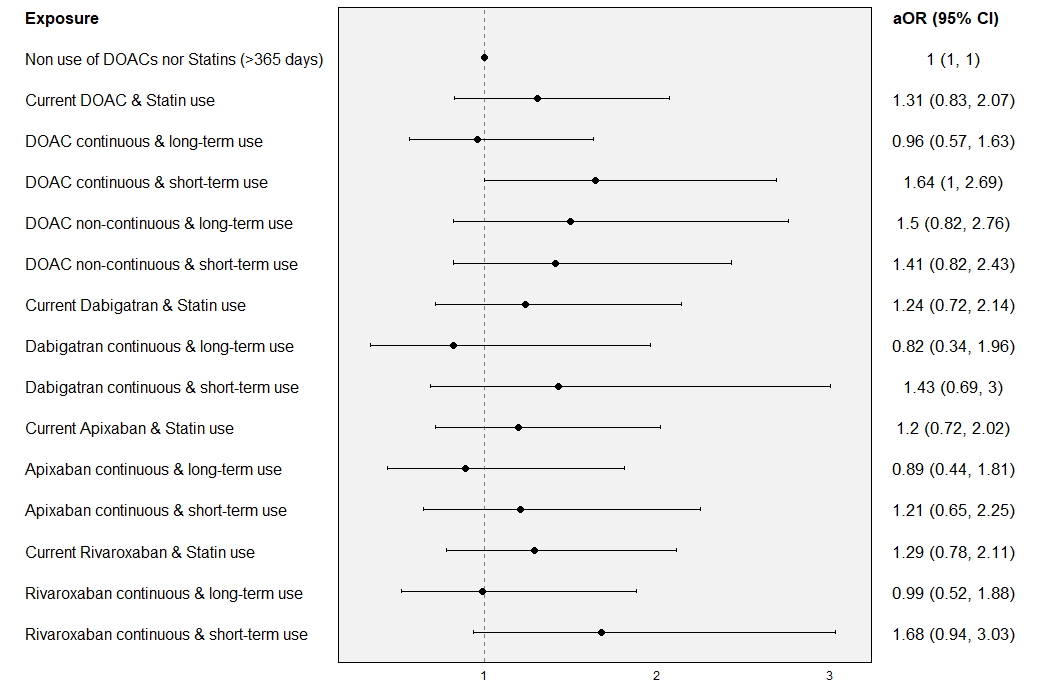

Supplement: Supplementary file 3 — Figure S3: Association between the concomitant use of DOACs and statins and the risk of major bleeding according to treatment duration, stratified by type of current use. [file PDS-34-e70245-s001.png]

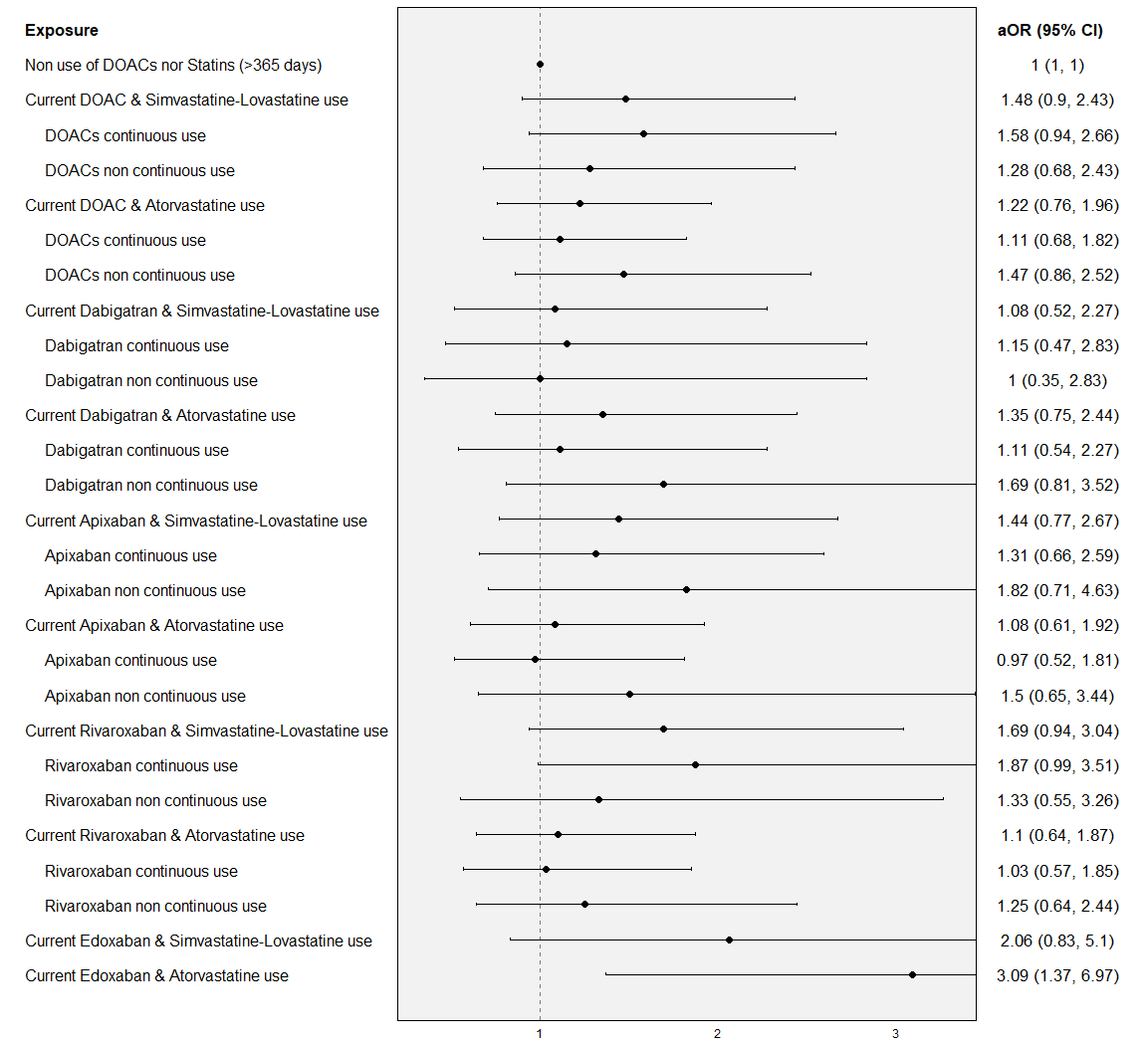

Supplement: Supplementary file 4 — Figure S4: Association between the concomitant use of DOACs and simvastatin/lovastatin or atorvastatin and the risk of major bleeding according to type of current use. [file PDS-34-e70245-s006.png]

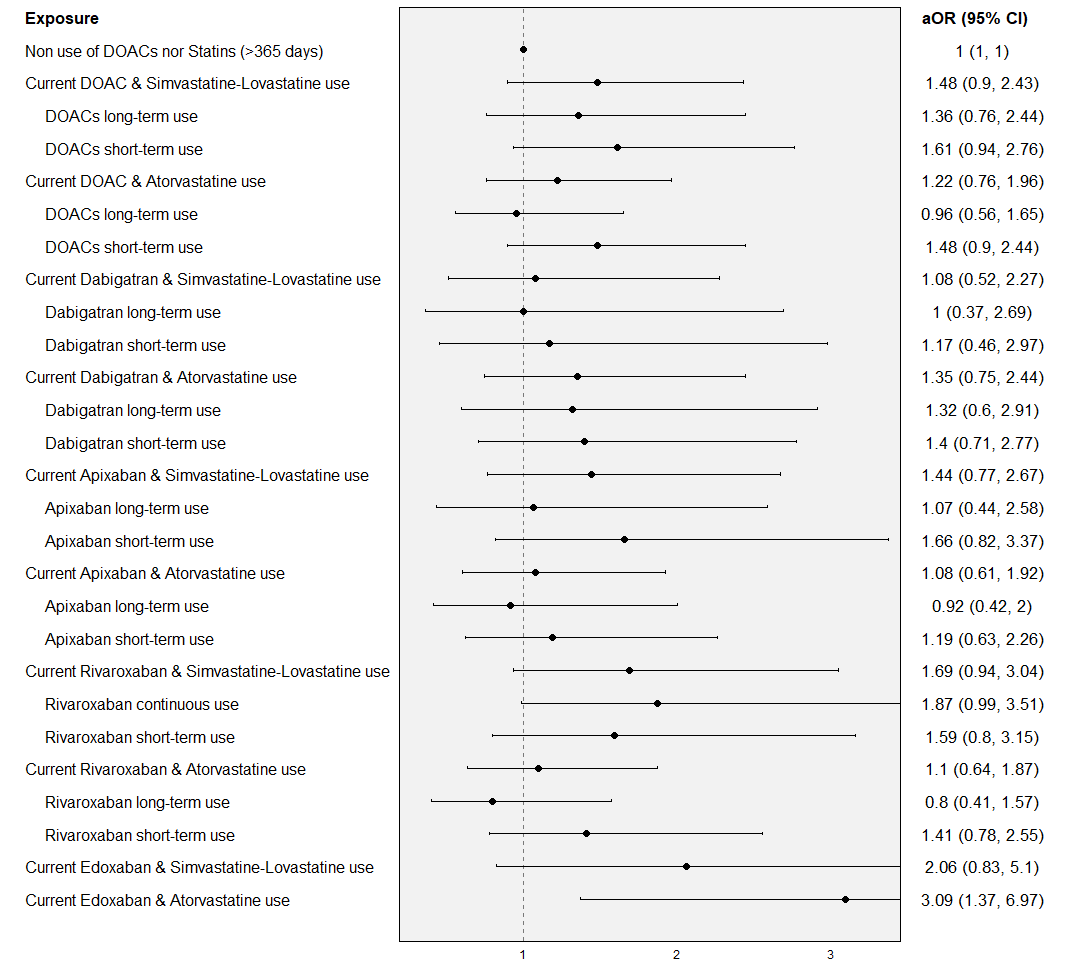

Supplement: Supplementary file 5 — Figure S5: Association between the concomitant use of DOACs and simvastatin/lovastatin or atorvastatin and the risk of major bleeding according to treatment duration. [file PDS-34-e70245-s003.png]

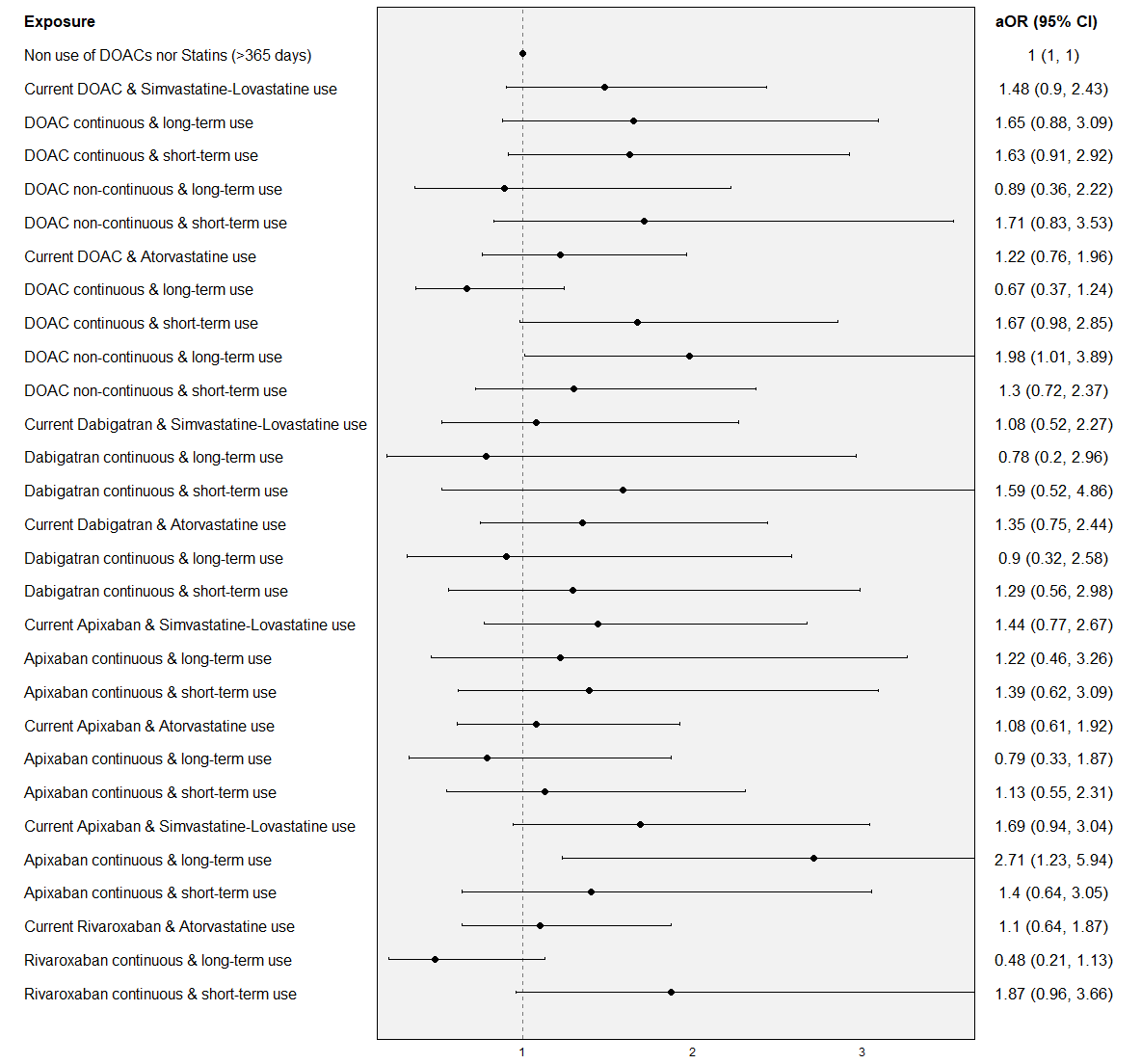

Supplement: Supplementary file 6 — Figure S6: Association between the concomitant use of DOACs and simvastatin/lovastatin or atorvastatin and the risk of major bleeding according to treatment duration, stratified by type of current use. [file PDS-34-e70245-s002.png]

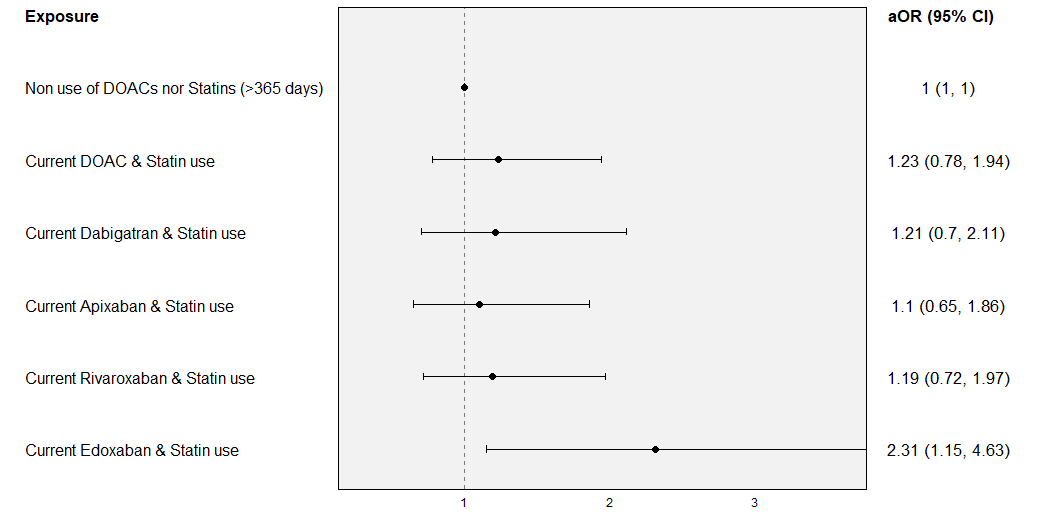

Supplement: Supplementary file 7 — Figure S7: Sensitivity analysis: Association between the concomitant use of DOACs and statins and the risk of major bleeding according to current use definition of 7 days. [file PDS-34-e70245-s007.png]
